# Supplementary material for: Actin assembly and non-muscle myosin activity drive dendrite retraction in an UNC-6/Netrin dependent self-avoidance response
Source: PLoS Genet. 2019 Jun 20;15(6):e1008228. doi: 10.1371/journal.pgen.1008228 (PMC6605669; doi:10.1371/journal.pgen.1008228)
Supplement: S1 Methods — (DOCX) [file pgen.1008228.s001.docx]

**Supporting Methods**

**Airy Scan imaging and analysis.** Animals were immobilized with 15mM levamisole/0.05% tricaine and mounted on 10% agarose pads in M9 buffer. Z stacks were captured using a Plan Apo 63x, 1.40 NA objective on a Zeiss LSM880 inverted fluorescence microscope outfitted with Airy Scan technology (Carl Zeiss Microscopy). Images of PVD::LifeAct::GFP and PVD::mCherry::UNC-34 (Figure S3 A-B) were processed to achieve Airy Scan (1.7x) super resolution and cropped using Fiji/ImageJ.

**Imaging and quantification of self-avoiding 3° dendrites:**

**Condition 1**: (See Figure S4A-C) L3 animals of strain CLP928 *twnEx382[Pser2.3::LifeAct::EGFP, Pser2.3::mCherry, Pgcy-8::gfp]*  [39] were immobilized for time-lapse imaging with 1 µl of agarose beads (Polybead carboxylate microspheres, Polysciences, Catalog # 15913-10, 0.05 µm) and mounted on a 10% agarose pad. Z-stacks of PVD dendrites (0.5 um depth, 5 slices) were imaged at both 35 second intervals with a 40X objective in a Nikon spinning disk confocal microscope. Contact between the 3° dendrites was established as a function of max feret (maximum distance between 2 points). Max feret (in pixels) was measured in the mCherry channel (cystosolic marker) with NIS elements and contact defined as the time-point at which the max-feret approaches zero. LifeAct::GFP and mCherry signals were measured for a ROI of 2 µm at the tips of the left and right dendrites in a single Z plane. Left and right fluorescence intensities were summed for comparison to a control ROI (2 µm) in an adjacent non-contacting region. The change in fluorescence intensity (∆F) for each contact and control region was calculated as the difference between the signal at contact (F_t_) and the fluorescence value at two time points prior to contact (F_t-2_) normalized to F_t-2_ or ∆F = (F_t_ - F_t-2_)/F_t-2_ [39]. These values were computed for contact and control ROIs for LifeAct::GFP and mCherry. Statistical tests used for comparison between the datasets (LifeAct::GFP contact vs control, mCherry contact vs control, LifeAct::GFP contact vs. mCherry contact) were 2-way ANOVA with Bonferroni correction and multiple t-tests with a False Discovery Rate of 1%. Both these tests yielded similar results. No significant differences were detected in the LifeAct::GFP signal in contacting vs non-contacting regions of 3° dendrites.

**Condition 2**: Late L3 larvae of P*F49H12.4::LifeAct::GFP; PF49H12.4::mCherry* were immobilized as above for imaging in the spinning disk confocal microscope. Z-stacks of PVD dendrites were imaged at either 1 or 1.30 minute intervals with a 100X objective (See Figure S4D). Contact is defined by the point at which the max feret (maximum distance between 2 points) between adjacent PVD 3º dendrites in the mCherry channel approached zero. For comparisons of LifeAct::GFP signals at the tips of 3º dendrites before and during contact, fluorescence intensities were determined from a 5 µm ROI centered at the point of contact and from a contiguous control (non-contact) 5 µm ROI positioned either to the left or right of the contact region (Figure 6F). The change in fluorescence intensity (∆F) for each contact and control region was calculated as the difference between the signal at contact (F_t_) and the fluorescence value at two time points prior to contact (F_t-2_) normalized to F_t-2_ or ∆F = (F_t_ - F_t-2_)/F_t-2_ [39]. For all measurements, the cytoplasmic mCherry marker was monitored to measure the Max Feret (in this case, maximum distance between two points) between the tips of adjacent 3º dendrites and the point of contact determined when the Max Feret approaches zero. Statistical comparisons (N = 8) were performed with a 2-way ANOVA with Bonferroni correction (Figure S4D). For representative traces shown in Figure S4, Nikon NIS Elements was used to obtain LifeAct::GFP and mCherry fluorescence measurements from a 1 µm ROI at the tips of the left and right 3º dendrites undergoing contact. Fluorescence intensity measurements of LifeAct::GFP and mCherry for the opposing dendrites were normalized against the maximum value for each fluorophore and plotted vs time.
