# Supplementary figures and images for: Actin assembly and non-muscle myosin activity drive dendrite retraction in an UNC-6/Netrin dependent self-avoidance response
Source: PLoS Genet. 2019 Jun 20;15(6):e1008228. doi: 10.1371/journal.pgen.1008228 (PMC6605669; doi:10.1371/journal.pgen.1008228)

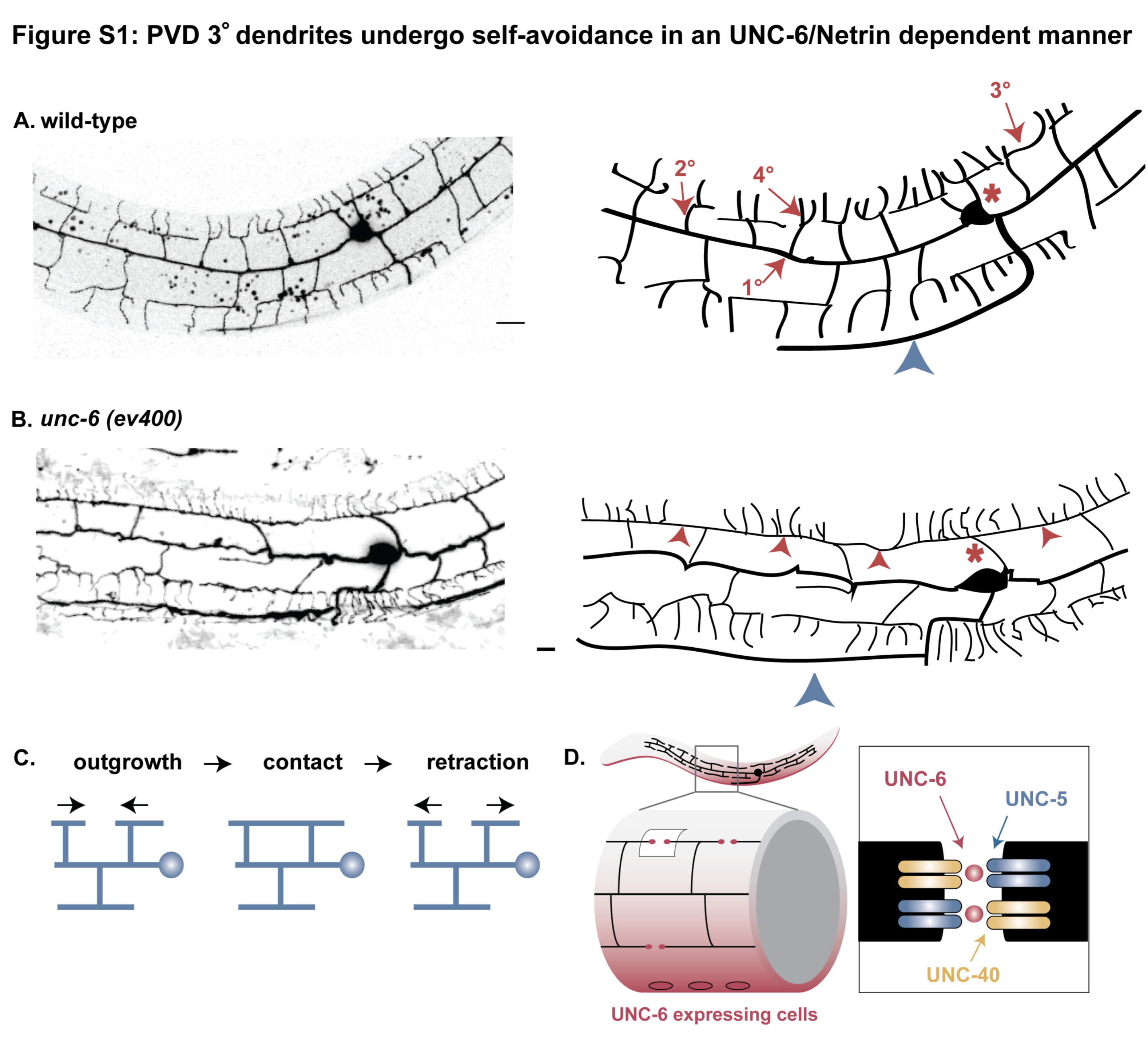

Supplement: S1 Fig — (A-B) Representative images and tracings of PVD sensory neurons in wild-type (A) and unc-6 (B) L4-stage larvae. PVD morphology was visualized with PVD::GFP or PVD::mCherry markers. PVD cell body marked with an asterisk. (A) 1°, 2°, 3° and 4° dendritic branches and single axon (blue arrowhead) are denoted. (B) Red arrowheads point to overlaps between adjacent 3° dendrites that failed to self-avoid in unc-6 mutants. Scale bars are 5 μm. C) Summary of self-avoidance in 3° PVD dendrites denoting growth, contact and retraction. (D) Model of self-avoidance mechanism involving reciprocal contact-dependent repulsion mediated by UNC-6/Netrin and its receptors, UNC-40/DCC and UNC-5 [9]. (TIFF) [file pgen.1008228.s002.tiff]

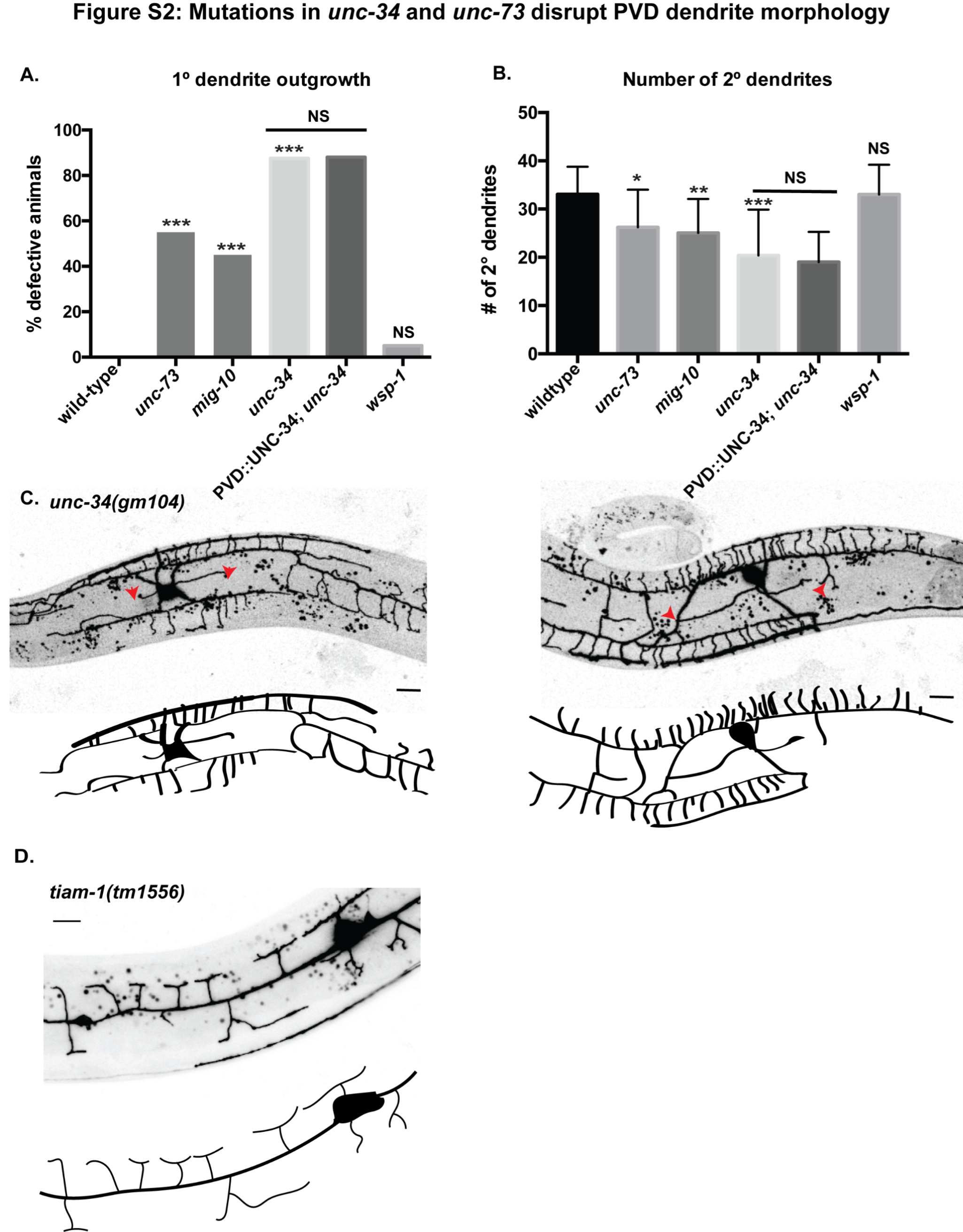

Supplement: S2 Fig — Quantification of 1° dendrite outgrowth and (B) number of 2° dendrites in wild-type, unc-34, unc-73, wsp-1 and PVD::mCherry::UNC-34;unc-34. Defective 1° dendrites show altered alignment and/or extension defects. Mutations in unc-34 and unc-73 but not wsp-1 result in defective 1° dendrite outgrowth and fewer 2° dendrites. Note that expression of UNC-34::mCherry in PVD restores 3° self-avoidance (Fig 3C) but does not rescue 1° and 2° dendritic defects which suggests that UNC-34 function could be required in other cell types for normal PVD 1° and 2° outgrowth, *p = 0.02, **p = 0.003, ***p<0.001, Fisher’s exact and 2-way ANOVA with Tukey’s correction for multiple comparison. (C) Representative images of PVD morphological defects in an unc-34 mutant. Note premature termination of 1° dendrites (red arrowheads). (D) Representative image and drawing of PVD branching defects in a tiam-1/GEF mutant at the L4 stage. Scale bars are 10 μm. Mutant alleles are unc-34(gm104), unc-73(rh40), wsp-1 (gm324) and tiam-1(tm1556). (TIFF) [file pgen.1008228.s003.tiff]

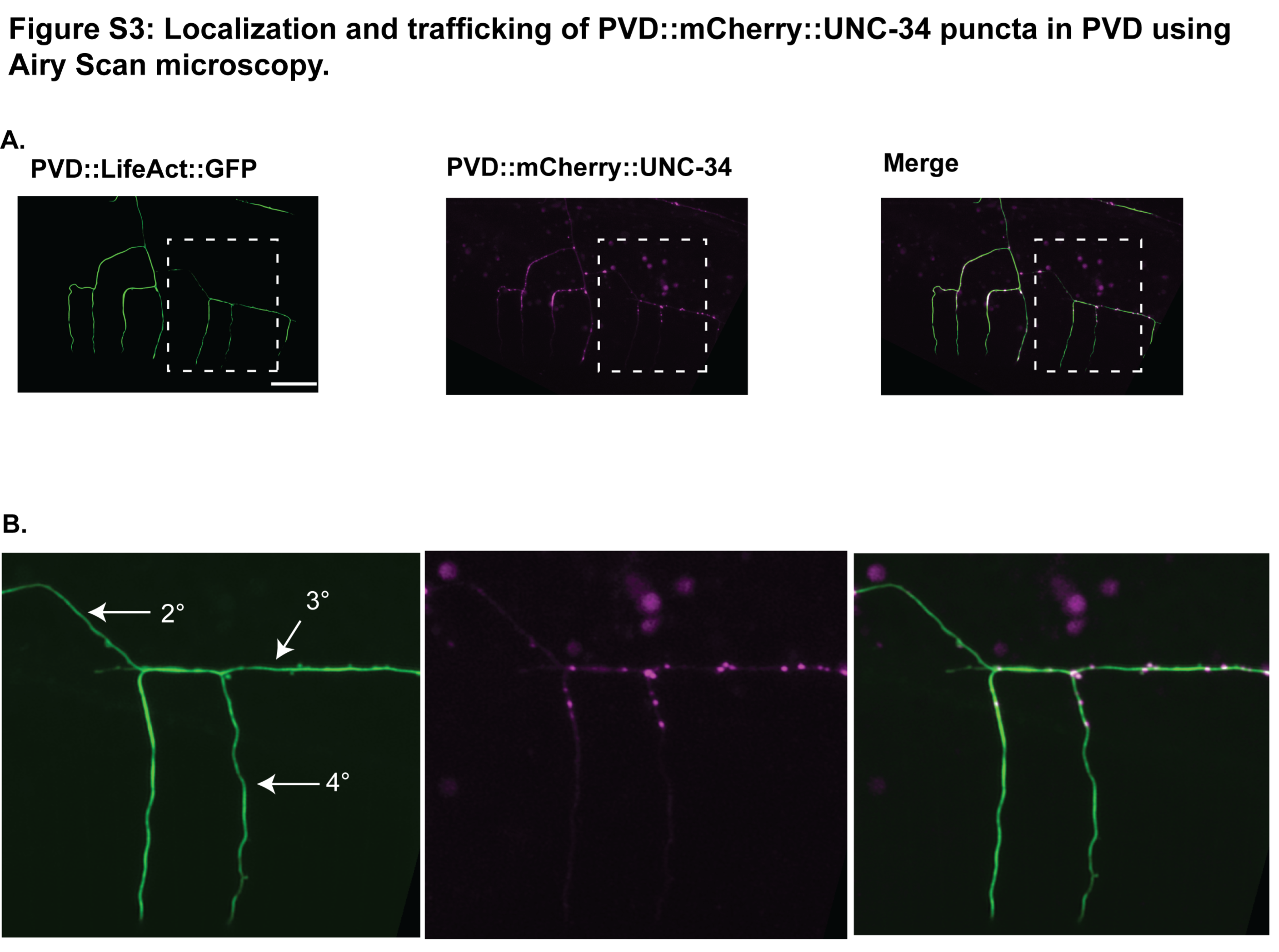

Supplement: S3 Fig — (A) Zeiss Airyscan images of PVD simultaneously labeled with PVD::LifeAct::GFP (green) and PVD::mCherry::UNC-34 (Magenta). Merge shown on right. Insets denote PVD dendrites (B). White arrows indicate 2°, 3°, and 4° dendrites. Scale bar is 10 μm. (TIFF) [file pgen.1008228.s004.tiff]

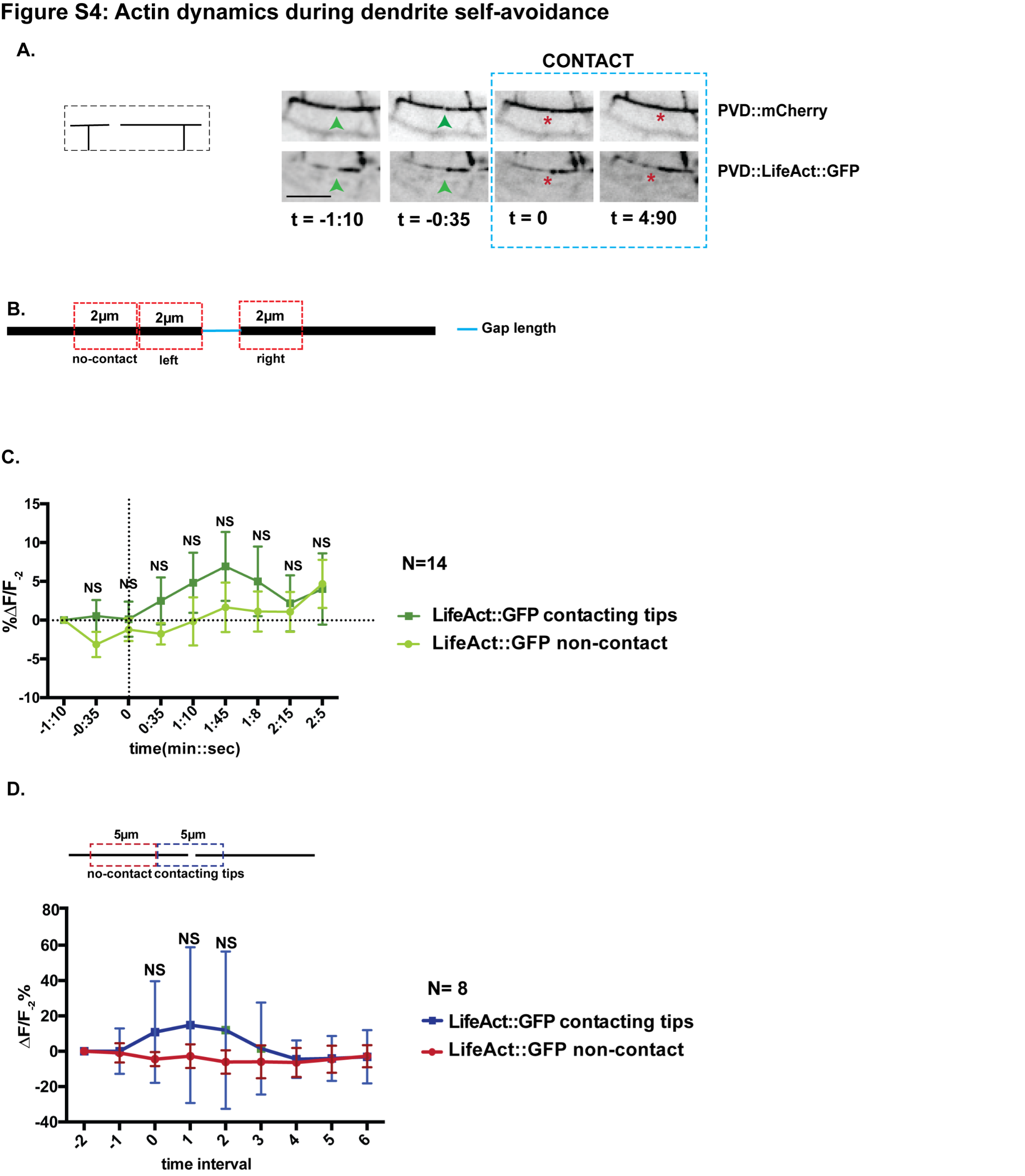

Supplement: S4 Fig — Schematic (left) of adjacent 3° dendrites and representative time lapse series (35 sec intervals) of PVD::mCherry and PVD::LifeAct::GFP in 3° dendrites showing self-avoidance response (Condition 1). Point of contact is indicated by an asterisk and period of contact denoted with dashed blue outline. Scale bar is 10 μm. (B) Schematic depicting ROIs for fluorescence intensity measurements at the tips of adjacent (left vs right) 3° dendrites that undergo contact and at an adjacent non-contact (control) region. (C) Graphical representation of the change in fluorescence intensity at each time point vs that of 2 time intervals (t-2) before contact (t = 0); Measurements were normalized to fluorescence intensity at t-2 or (Ft-2) [39]. Normalized fluorescence intensity values were plotted against time (min) for LifeAct::GFP contacting 3° dendrites vs non-contact, and were compared by 2-way Anova with Bonferonni correction, p = 0.54 (N = 14). (D) Time lapse series collected with 100X objective at 1–1.3 minute intervals (N = 8) (Condition 2). The values for LifeAct::GFP at contact vs non-contacting region of 3° dendrites at each time-point were compared by 2-way Anova with Bonferonni correction, p = 0.74, NS (Not Significant). (TIFF) [file pgen.1008228.s005.tiff]

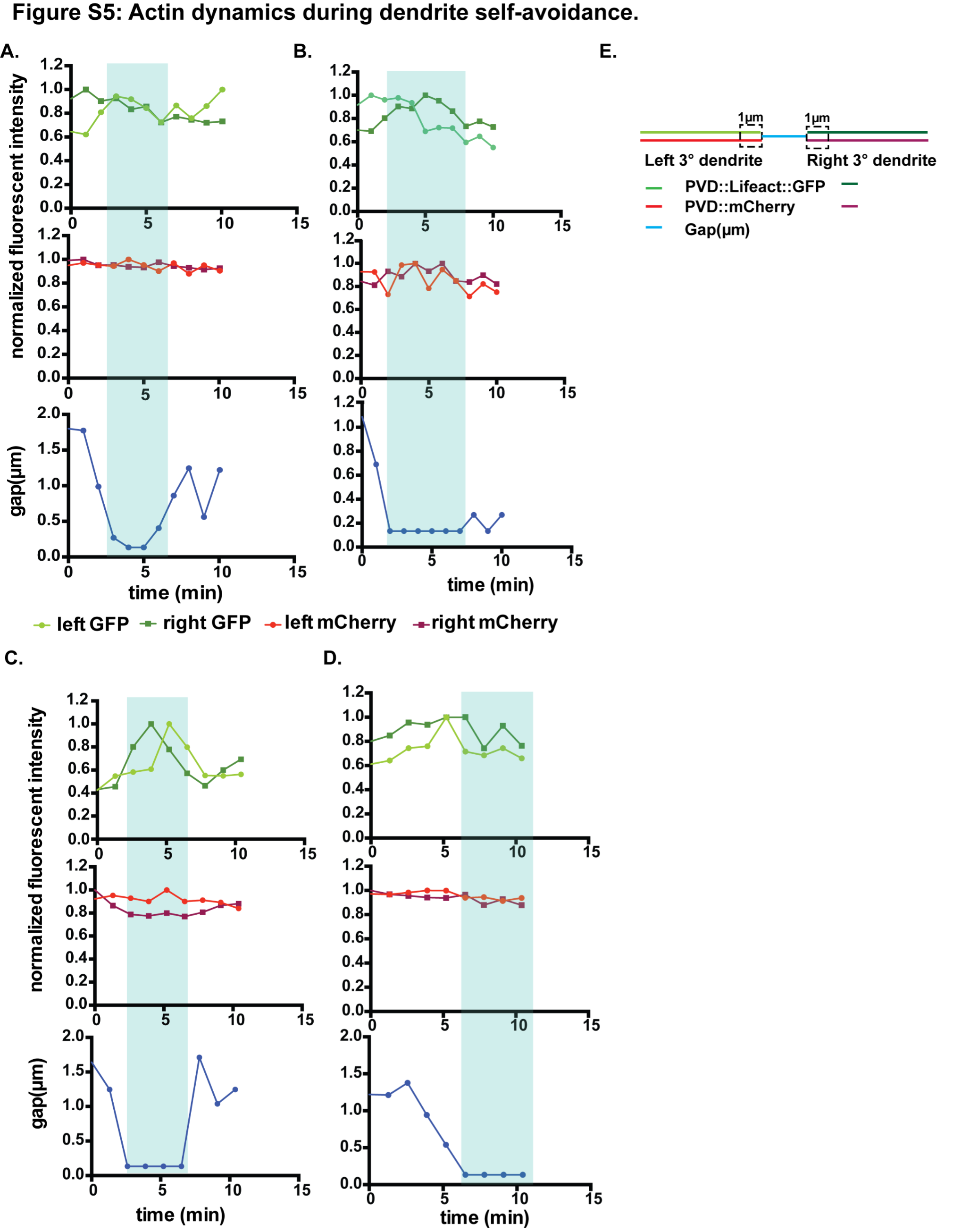

Supplement: S5 Fig — (A-D) Fluorescent intensity traces wild-type PVD dendrites during 3° dendrite self-avoidance. Fluorescent intensity measurements of PVD::LifeAct::GFP and PVD::mCherry were acquired at either 1 min (A-B) or 1.3 min (C-D) intervals from a 1μm region at the tips of the growing and contacting left and right 3° dendrites (Late L3-L4 animals). Measurements were normalized against the maximum intensity during a ~10 minute interval that includes at least one contact event. The gap between the left and right 3° dendrites undergoing contact was determined from the cytoplasmic mCherry signal and plotted against time. The period of contact (vertical shading) corresponds to a minimum value for the gap (0–0.14μm) between contacting left and right 3° dendrites. (E) Representation of 3° dendrites with color schemes to indicate left and right 3° dendrites and 1μm regions quantified. (TIFF) [file pgen.1008228.s006.tiff]
